# Supplementary material for: Gene augmentation prevents retinal degeneration in a CRISPR/Cas9-based mouse model of PRPF31 retinitis pigmentosa
Source: Nat Commun. 2022 Dec 13;13:7695. doi: 10.1038/s41467-022-35361-8 (PMC9744804; doi:10.1038/s41467-022-35361-8)
Supplement: Supplementary file 1 — Supplementary Information [file 41467_2022_35361_MOESM1_ESM.pdf]

Supplementary figures

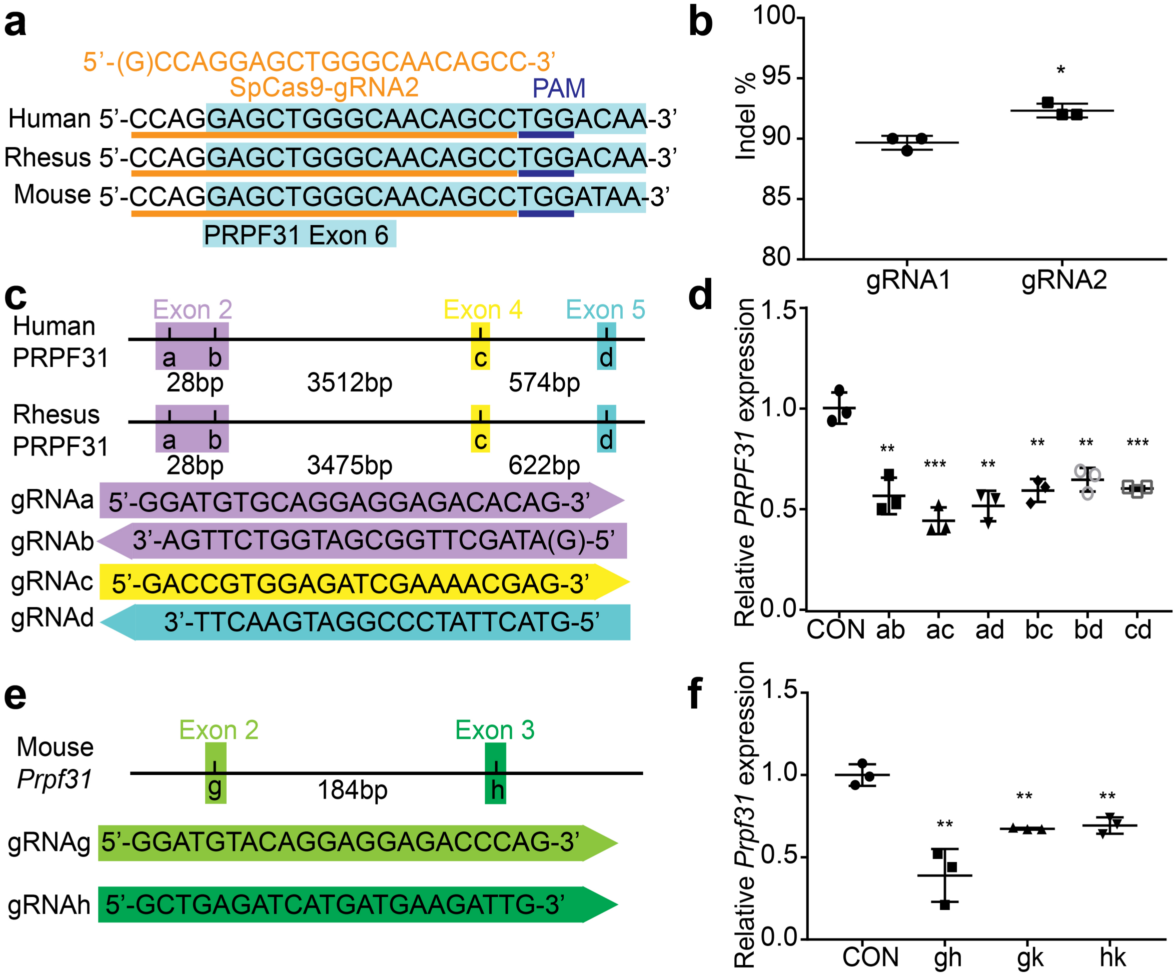

Supplementary Fig.1 gRNA screening**. a** Schematic representation of the location of SpCas9 gRNA2 target in human, macaque, and mouse *PRPF31* exon 6. **b** Indel% of *PRPF31* in the SpCas9 and gRNA1-GFP or gRNA2-GFP plasmid co-transfected and FACS-sorted GFP positive cells (n=3). **c** Schematic representation of the location of SaCa9 gRNAa/b/c/d targets in human and rhesus *PRPF31.* The distance of each gRNA stated in bp were counted according to the predicted cut site (3bp before PAM). **d** Relative *PRPF31* mRNA expression level from SaCas9 gRNAa/b/c/d co-transfected HEK293T cells (n=3). **e** Schematic representation of the location of SaCas9 gRNAg/h targets in mouse *Prpf31.* **f** Relative *PRPF31* mRNA expression level of SaCas9 gRNAg/h/k co-transfected NIH/3T3 cells (n=3). CON: control samples transfected with plasmid CAG-GFP. Data were shown as means ± SEMs and analyzed with unpaired two-tailed t-test. *P < .05; **P < .01; *** P < .001.

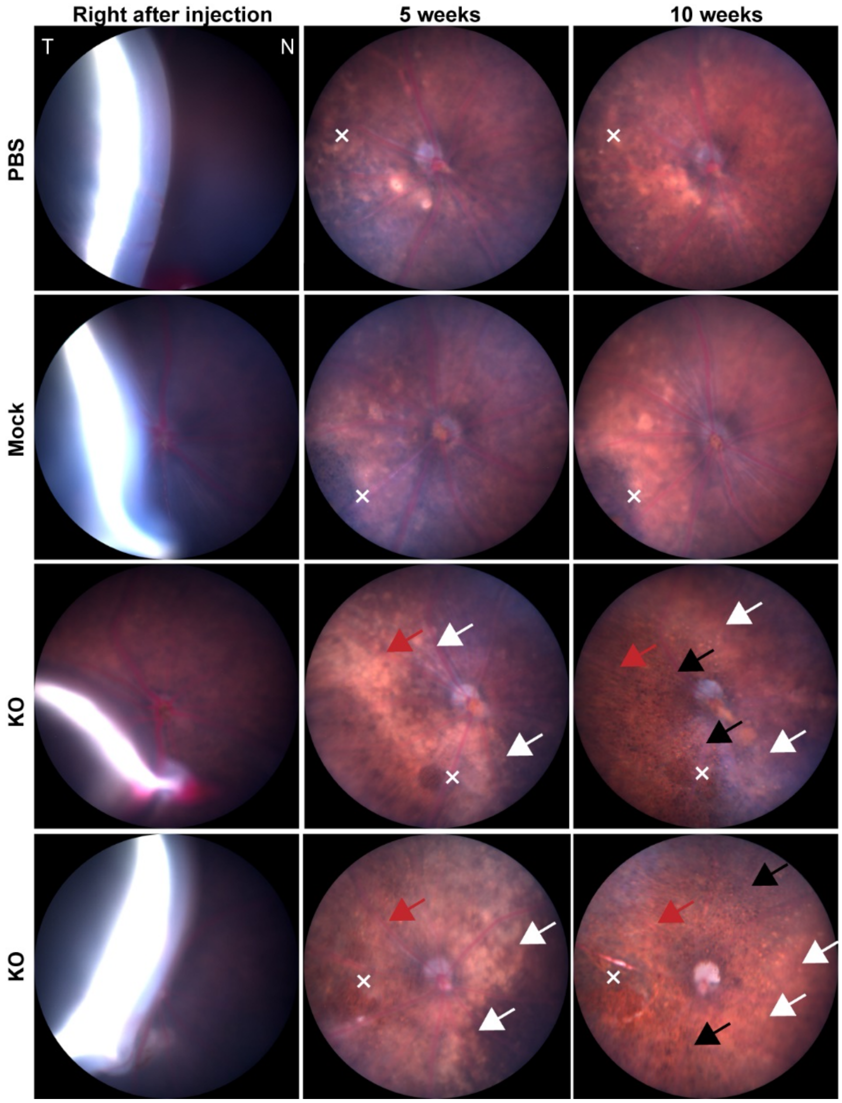

Supplementary Fig. 2 Fundus images taken following subretinal injections of *Prfpf31-KO* vectors to WT mice. Images were collected immediately after injection, in order to record the location of the injection site and presence of the bleb, and 5 weeks and 10 weeks p.i.. The injection site (×) was made at the temporal retina, with a 1.5ul injection volume, with the resulting bleb covering almost half of the retina. At 5 weeks p.i., retinal pallor (white arrows) was found in the temporal retina of the KO eyes, which corresponded to the coverage of the injection bleb. At 10 weeks p.i., punctate black pigmentation (black arrows) formed in the area of retinal pallor in KO eyes, and thinning of blood vessels was also observed (red arrows). (n=16 for all groups.)

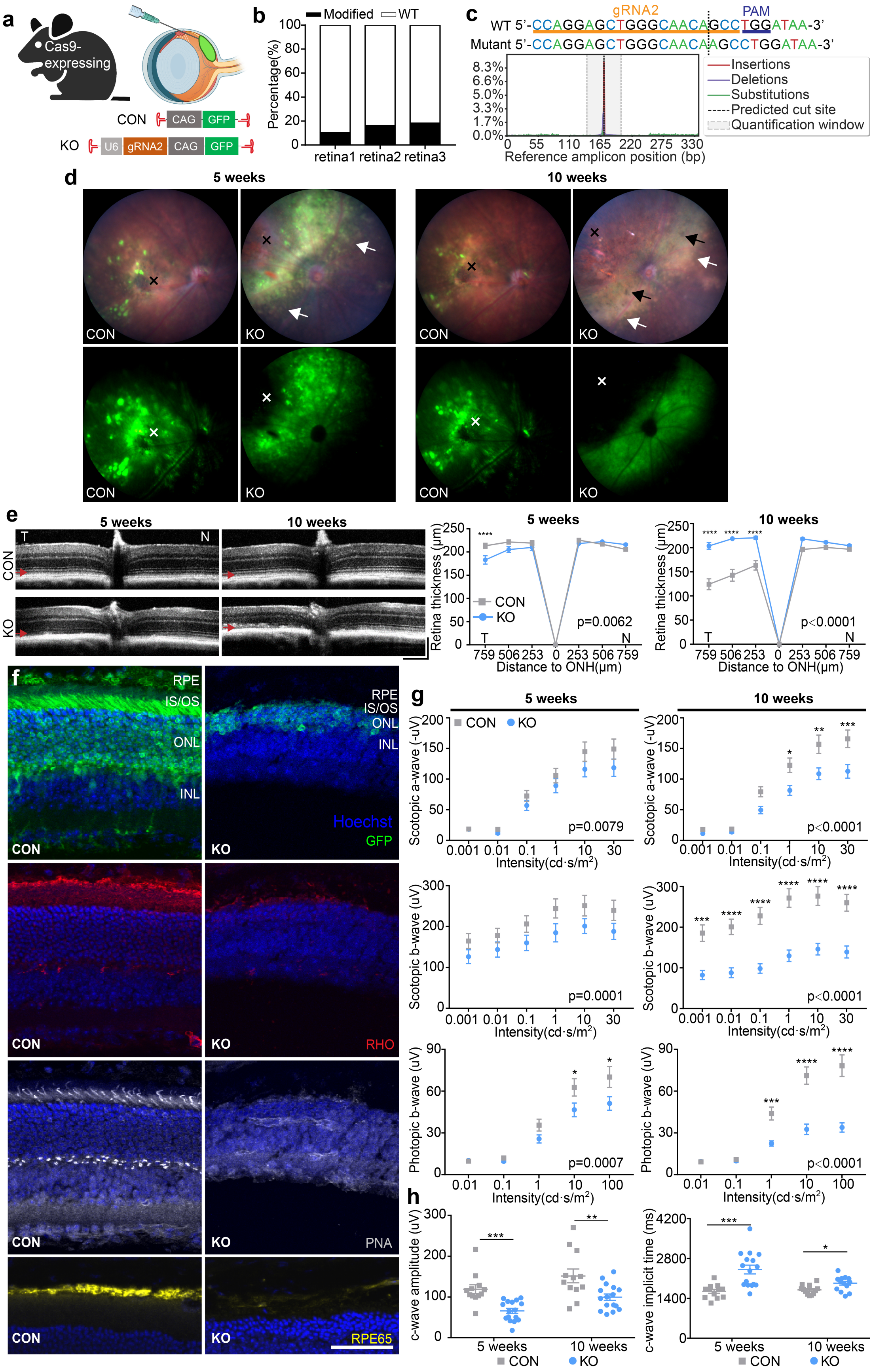
a
Cas9-expressing
CON
CAG
GFP
KO
U6
gRNA2
CAG
GFP
b
Modified
WT
Percentage(%)
100
80
60
40
20
0
retina1 retina2 retina3
c
gRNA2
PAM
WT 5'-CCAGGAGCTGGGCAACAGCCTGGATAA-3'
Mutant 5'-CCAGGAGCTGGGCAACAAGCCTGGATAA-3'
8.3%
6.6%
5.0%
3.3%
1.7%
0.0%
0 55 110 165 220 275 330
Reference amplicon position (bp)
Insertions
Deletions
Substitutions
Predicted cut site
Quantification window
d
5 weeks
10 weeks
CON
KO
e
5 weeks
10 weeks
T
N
CON
KO
Retina thickness (μm)
250
200
150
100
50
0
p=0.0062
p<0.0001
759 506 253 0 253 506 759
Distance to ONH(μm)
f
RPE
IS/OS
ONL
INL
Hoechst
GFP
RHO
PNA
RPE65
g
Scotopic a-wave (-uV)
Scotopic b-wave (uV)
Photopic b-wave (uV)
Intensity(cd·s/m²)
0.001 0.01 0.1 1 10 30
0.01 0.1 1 10 100
p=0.0079
p<0.0001
p=0.0001
p<0.0001
p=0.0007
p<0.0001
h
c-wave amplitude (uV)
c-wave implicit time (ms)
300
200
100
0
4200
2800
1400
0
5 weeks
10 weeks

Supplementary Fig. 3 Subretinal injection of a *Prpf31*-KO vector results in structural and functional degeneration in outer retina. a Illustration of experimental approach. KO vector 7m8-U6-gRNA2-CAG-GFP (0.6 µl, 2.13E+12 vg/ml) was subretinally delivered to SpCas9-expressing mice, and the control vector 7m8-CAG-GFP (0.6 µl, 2.46E+12 vg/ml) was delivered to the contralateral eye. **b** Deep sequencing of DNA from whole retina of the KO eyes 5 weeks p.i. revealed 14.92±4.10% editing of *Prpf31* at the gRNA2 target region (n=3). **c** Mutations in edited *Prpf31*. The main editing event identified was the insertion of an adenosine at the predicted cut site (3bp before the PAM) (n=3). **d** Fundus images. Injections were made at the temporal retina (×). At 5 weeks p.i., retinal pallor (white arrows) was observed in KO eyes near the injection site, corresponding to the area of highest GFP expression. At 10 weeks p.i., the area of retinal pallor expanded, and black-brownish deposits (black arrows) were observed. GFP expression disappeared near the injection site over time, indicating the degeneration of *Prpf31*-KO cells (CON n=17, KO-Rescue n=23). **e** OCT of retina and quantification of total retina thickness (neural retina and RPE) (n=7). Red arrows, IS/OS junction. ONH, optic nerve head; T, temporal; N, nasal. Scale bar vertical, 100 µm; Scale bar horizontal, 200 µm. **f** Retinal cross sections. GFP (green) was expressed in all retina layers with strongest expression in the outer retina. KO eyes had a significantly thinner ONL, with notably reduced rods (RHO in red), cones (PNA in grey) and RPE markers (RPE65 in yellow). Scale bar, 50µm. **g** Quantification of ERG scotopic and photopic a- and b-wave amplitudes (n=12). **h** Quantification of dark-adapted c-wave amplitudes and implicit time (KO n=16, Control n=12). Data were shown as mean ± SEM. Significance of the overall treatment factor was analyzed using two-way ANOVA and stated as a P value in the plots. Significance of treatment factor at each distance from ONH (for OCT) or each flash intensity (for ERG) was calculated by Sidak multiple comparisons test and marked as *P < .05; **P < .01; *** P < .001; **** P < .0001.

Supplementary Table 1 P-values from gene expression analysis

	Prpf31		Vim		Gfap		Casp1		Casp4		Casp12		Rho		Opn1sw		Rpgr	
**weeks**	log2(FC)	P-value	log2(FC)	P-value	log2(FC)	P-value	log2(FC)	P-value	log2(FC)	P-value	log2(FC)	P-value	log2(FC)	P-value	log2(FC)	P-value	log2(FC)	P-value
**1**	-0.12982	0.19169	-0.12282	0.40214	-0.12111	0.35084	0.38826	0.00416	0.27775	0.00744	0.19742	0.08727	-0.09675	0.4608	0.1564	0.19607	0.08401	0.4279
**2**	0.09207	0.45132	0.3683	0.04162	-0.18565	0.22971	1.74601	2.6E-19	1.10929	2.2E-09	1.65712	2.7E-17	-0.04299	0.76783	0.17189	0.36761	-0.12129	0.41395
**3**	0.48322	8.4E-05	0.43686	0.01649	0.18739	0.24107	0.76849	1.7E-05	0.32637	0.01687	0.35686	0.04465	0.02766	0.85617	-0.53189	0.00339	0.07022	0.62539
**4**	0.44536	0.00268	0.67682	5.1E-06	0.19076	0.32412	0.3651	0.05961	0.25304	0.14551	0.93993	0.00033	0.27391	0.21608	-0.09106	0.72641	-0.18841	0.43487
**10**	-0.30898	0.01409	0.94426	6.5E-16	0.419	0.00027	3.55652	6.5E-31	2.27505	4.3E-06	4.18579	7.6E-32	-0.88892	8E-08	-1.48999	0.00106	-1.19307	7.2E-06

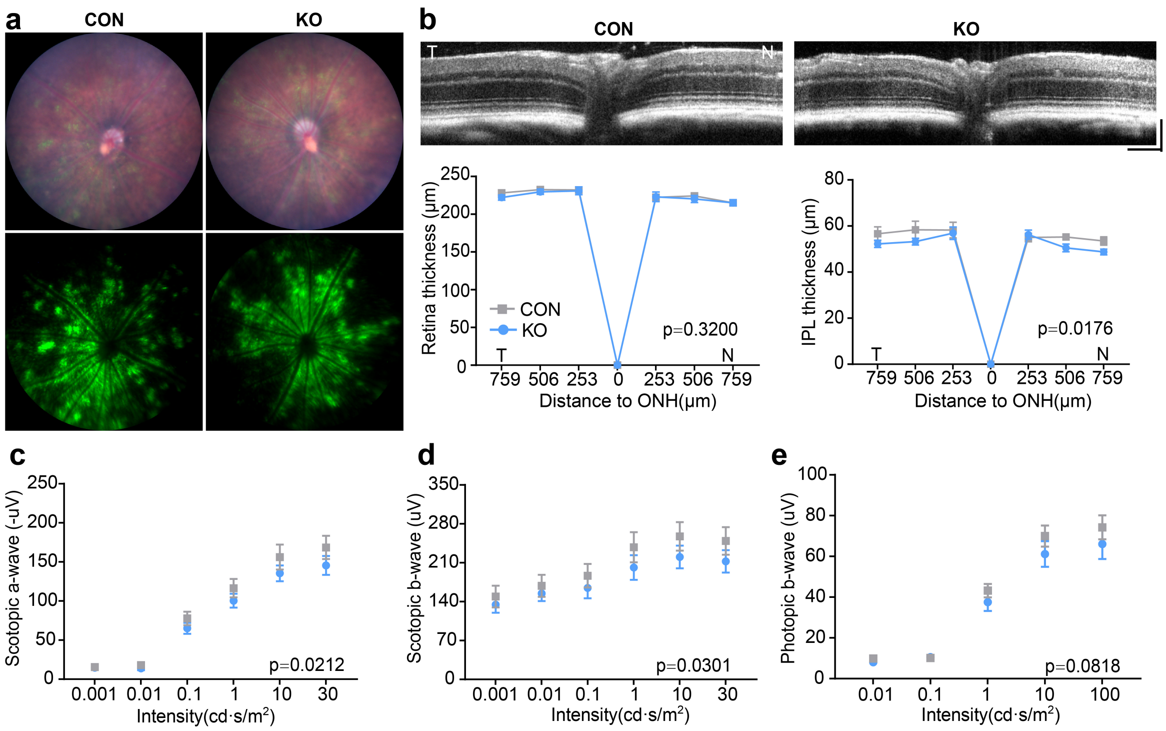

Supplementary Fig. 4 Retinal condition 5 weeks after intravitreal injection of *Prpf31*-KO vectors. Fig. 2 **Intravitreal injection of *Prpf31*-KO vector results in structural and functional changes primarily in the inner retina. a** Fundus images. GFP expression was observed across the expanse of the retina (CON n=19, KO n=19). **b** OCT of retina and quantification of total retinal thickness (neural retina and RPE) and IPL thickness (n=8). Scale bar vertical, 100 μm; Scale bar horizontal, 200 μm. **c-e** Quantification of scotopic and photopic a- and b-waves from ERG recordings (n=10). Data are shown as means ± SEMs and were analyzed using multiple comparisons and two-way ANOVA. Significance of the overall treatment factor was stated as a P value in the plots. The significance of the treatment factor at each distance from ONH (for OCT) or each flash intensity (for ERG) was calculated by Sidak multiple comparisons test and marked as *P < .05; **P < .01; *** P < .001; **** P < .0001. KO, knock out; CON, control. ONH, optic nerve head; T, temporal; N, nasal.

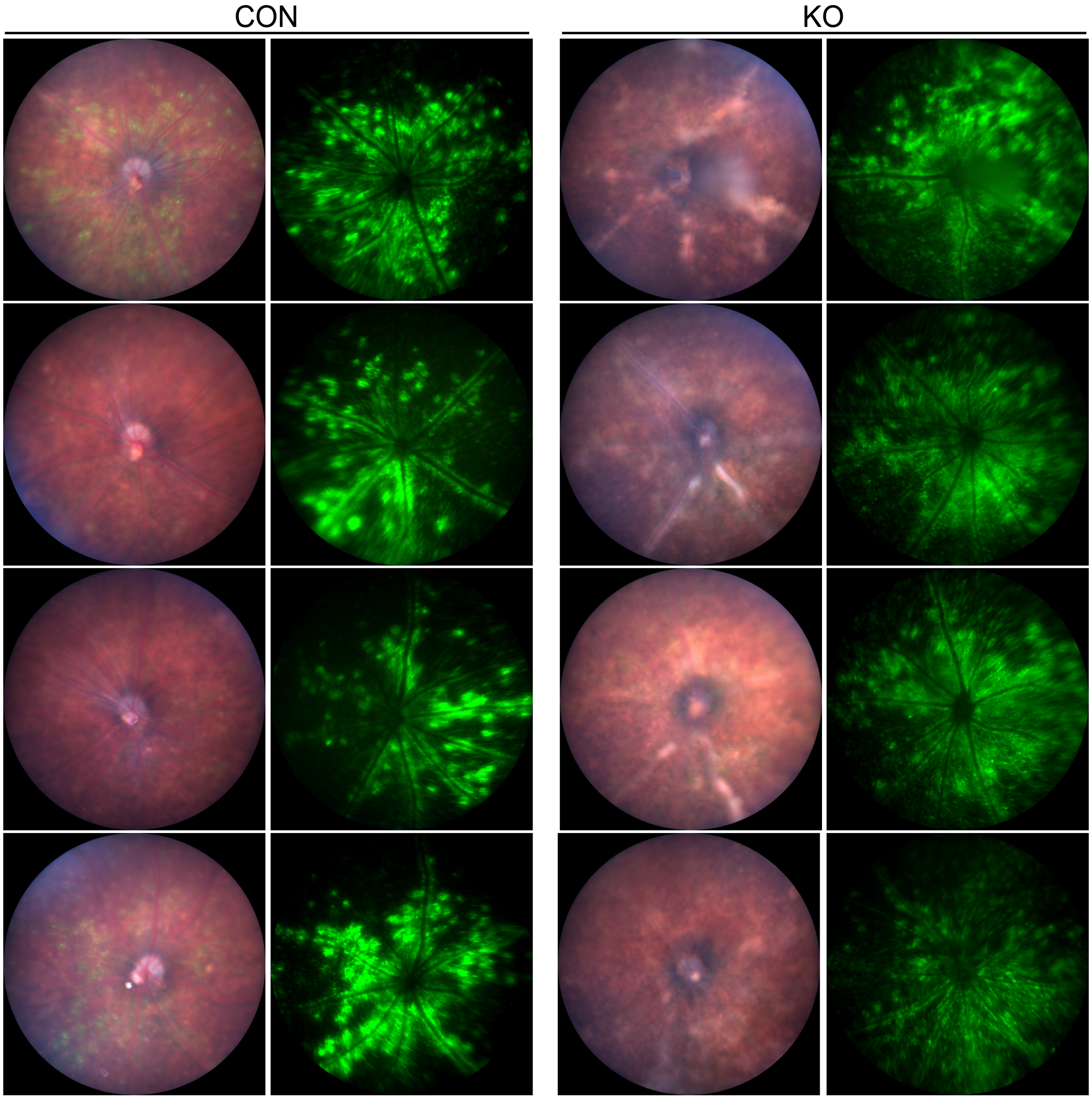

Supplementary Fig. 5 Fundus images 15 weeks after intravitreal injection of *Prpf31*-KO vector. KO eyes show retinal pallor alongside the blood vessels, corresponding with the area that has highest level of GFP expression. No retinal pallor was observed in the control eyes. (CON n=19, KO n=19.)

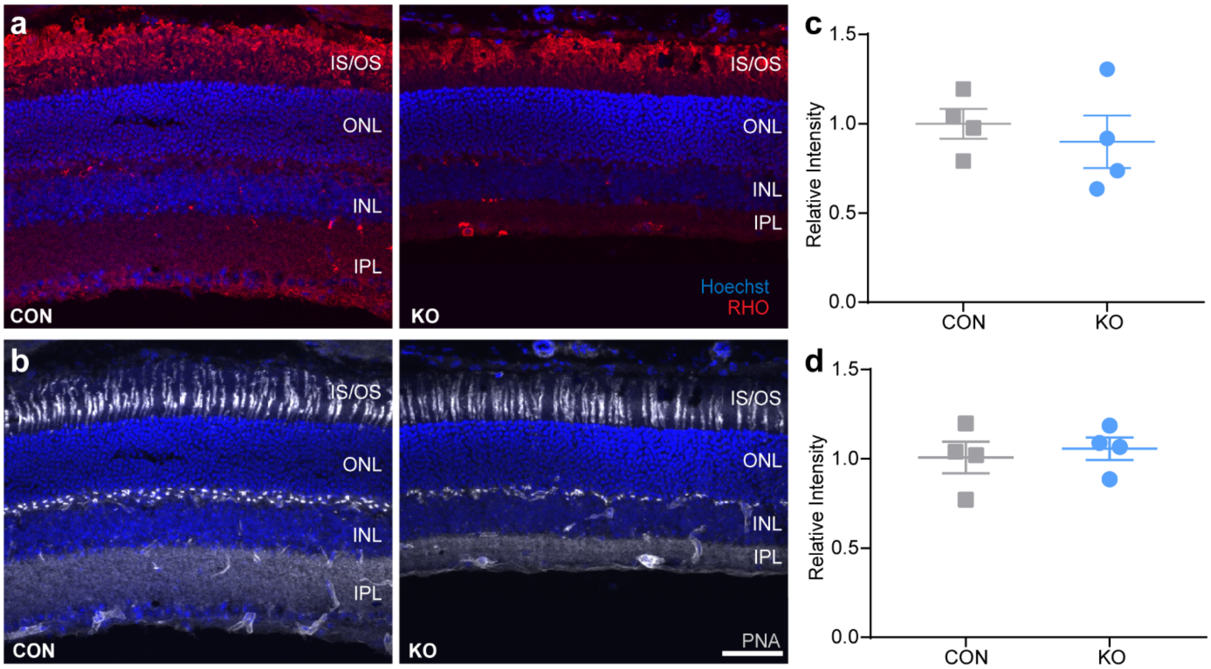

Supplementary Fig. 6 Retinal condition 15 weeks after intravitreal injection of *Prpf31*-KO vectors. a-b Retinal cross sections 15 weeks p.i.. Scale bar, 50 µm. **c** Quantification of a rod specific marker (RHO) by intensity (n=4). **d** Quantification of a cone specific marker (PNA) by intensity (n=4). No statistically significant difference was seen when comparing the KO eyes to the control eyes. Data were shown as mean ± SEM and were analyzed with an unpaired two-tailed t-test.

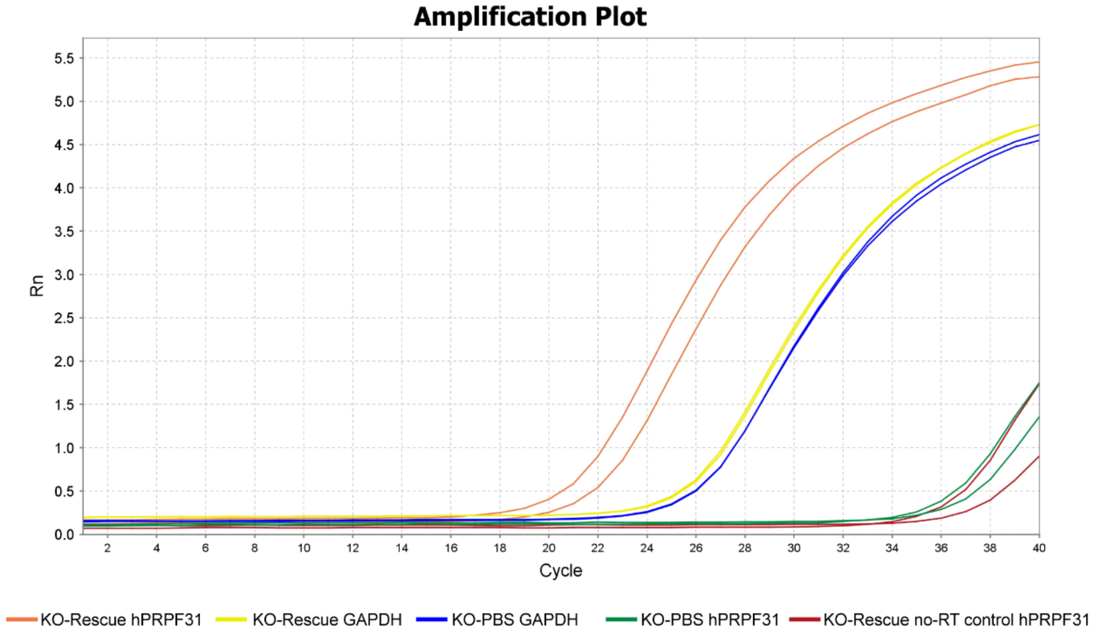

Supplementary Fig. 7 qRT-PCR confirms expression of hPRPF31 in KO-Rescue retinas. qRT-PCR amplification plot from KO-PBS and KO-Rescue retina using hPRPF31 specific primers. Successful expression of hPRPF31 was confirmed in the KO-Rescue retina cDNA samples (orange curve) with no expression of hPRPF31 detected in KO-PBS cDNA samples (green curve).

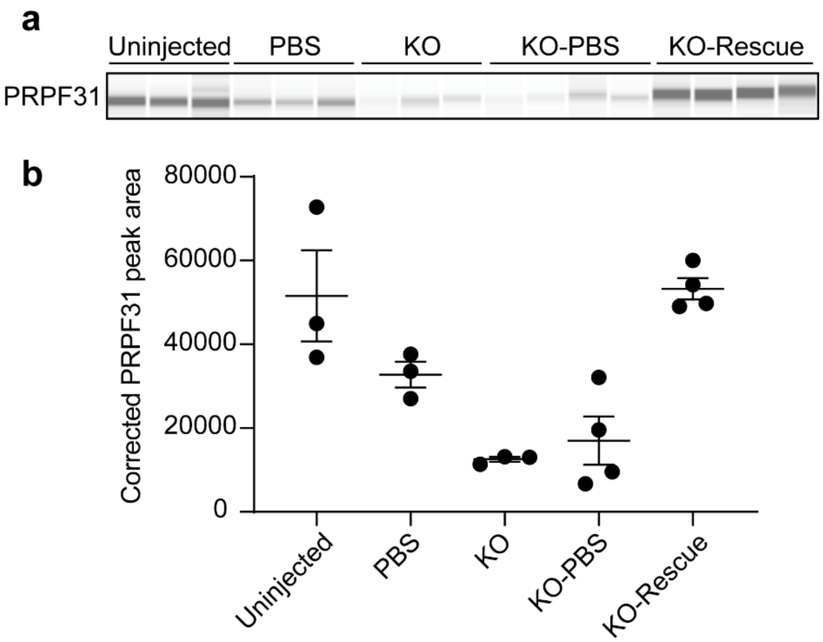

Supplementary Fig. 8 PRPF31 protein levels were reduced in KO eyes and were restored to normal levels in KO-Rescue eyes 10 weeks p.i.. Protein levels are shown for uninjected WT mice (Uninjected), PBS-injected eyes (1.5 µl PBS), KO vector-injected eyes (KO; 1.5 µl 7m8-CMV-SaCas9-U6-gRNAg/h, 1.01E+12 vg/ml), KO vector+PBS-injected eyes (KO-PBS; 0.75 µl PBS + 0.75 µl 7m8-CMV-SaCas9-U6-gRNAg/h, 1.01E+12 vg/ml), and KO vector+rescue vector-injected eyes (KO-Rescue; 0.75 µl 7m8-CMV-SaCas9-U6-gRNAg/h, 1.01E+12 vg/ml + 0.75 µl 7m8-CAG-hPRPF31,1.05E+12 vg/ml). Retinas were collected 10 weeks after injection. Automated quantitative Western blotting (Jess, ProteinSimple) was performed according to manufacturer's instructions, using the protein normalization module. Samples from individual biological replicates are shown. **a** PRPF31 protein levels in mouse retinal samples, visualized using automated Western blotting on the Jess system. **b** Quantification of protein levels (PRPF31 peak area), corrected and normalized to total protein content of individual samples (see source data). (Uninjected n=3, PBS n=3, KO n=3, KO-PBS n=4, KO-Rescue n=4. Data are shown as mean ± SEM.)

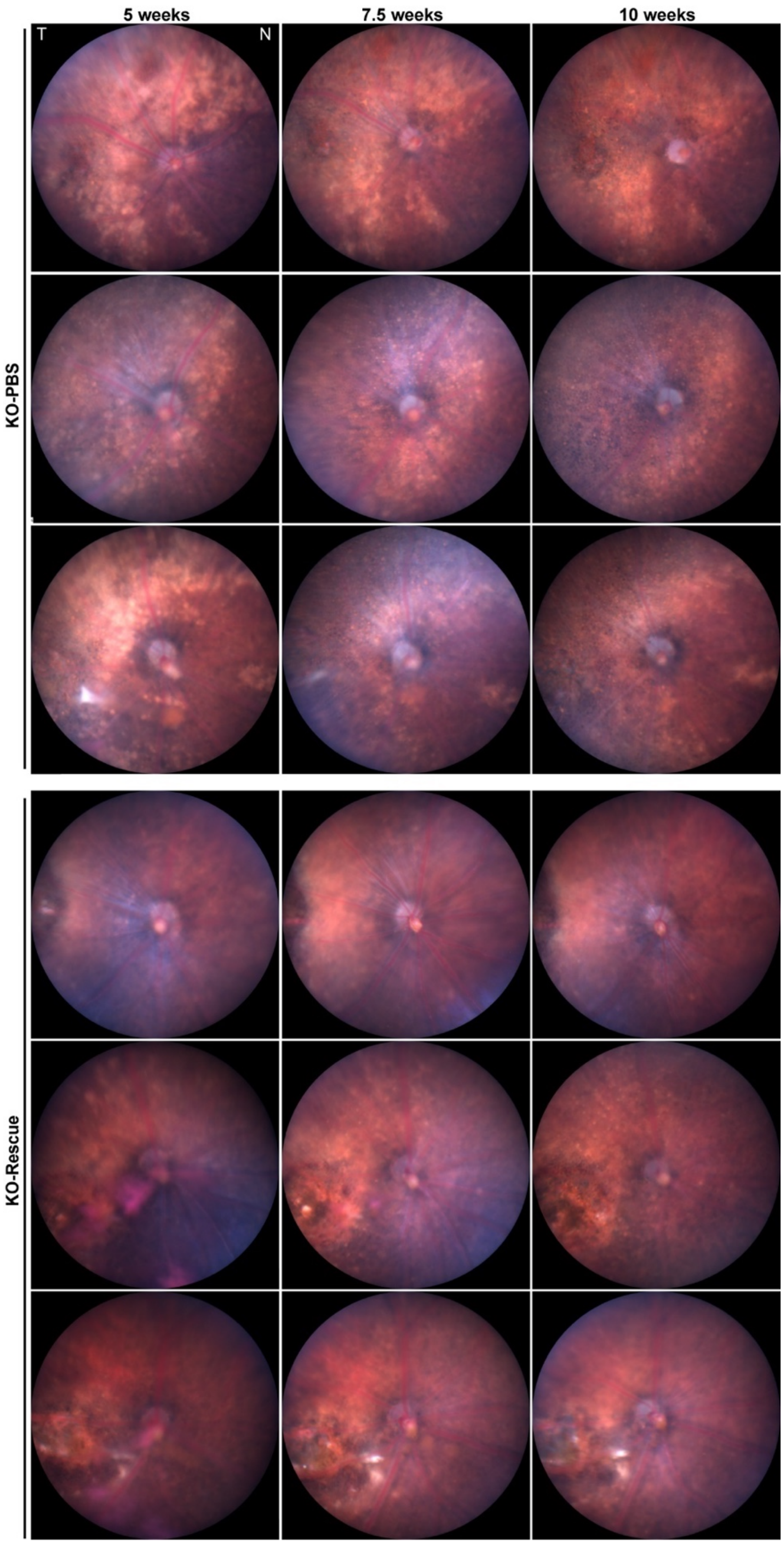

Supplementary Fig. 9 Fundus images acquired 5, 7.5 and 10 weeks p.i., from WT mice injected subretinally with KO-PBS and KO-Rescue vectors. (KO-PBS n=16, KO-Rescue n=16.)

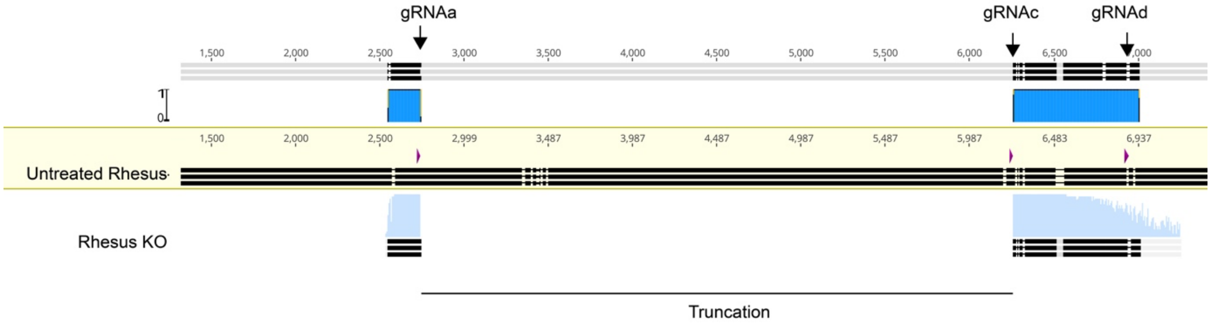

Supplementary Fig. 10 The truncation of DNA between gRNAa and gRNAc in rhesus retinal explants. Alignment of DNA from untreated and KO Rhesus retinas. The size of the DNA deletion corresponded to the length of sequence between gRNAa and gRNAc and was verified by Sanger sequencing, suggesting that gRNAa and gRNAc were more efficient than gRNAd.

Supplementary Table 2 gRNAs used in experiments

gRNA name	Sequences 5'-3'	Target
SpCas9-gRNA1	(G)CAGGCTGTTGCCCAGCTCCT	human, rhesus, mouse PRPF31 exon 6
SpCas9-gRNA2	(G)CCAGGAGCTGGGCAACAGCC	human, rhesus, mouse PRPF31 exon 6
SaCas9-gRNAa	GGATGTGCAGGAGGAGACACAG	Human, rhesus PRPF31 exon 2
SaCas9-gRNAb	(G)ATAGCTTGGCGATGGTCTTGA	Human, rhesus PRPF31 exon 2
SaCas9-gRNAc	GACCGTGGAGATCGAAAACGAG	Human, rhesus PRPF31 exon 4
SaCas9-gRNAd	GTACTTATCCCGGATGAACTT	Human, rhesus PRPF31 exon 5
SaCas9-gRNAg	GGATGTACAGGAGGAGACCCAG	Mouse PRPF31 exon 2
SaCas9-gRNAh	GCTGAGATCATGATGAAGATTG	Mouse PRPF31 exon 3
SaCas9-gRNAk	GTAATCCAGAGCATTGGGCACC	Mouse PRPF31 exon 5

Supplementary Table 3 Titers of viruses used in *ex vivo* human and primate retinal experiments

AAV	Titer (vg/ml)	Concentration of AAV in culture well (vg/ml)	Retina	Experiments
K912-CAG-GFP	2.17E+12	2.50E+10	rhesus	IHC section, DNA gel
K912-CMV-SaCas9-gRNAa/c/d	1.33E+12	2.04E+10		
K912-CAG-GFP	1.05E+12	2.63E+10	donor 1	DNA gel, WB
K912-CMV-SaCas9-gRNAa/c/d	1.78E+13	3.42E+11		
K912-CAG-GFP	2.32E+12	6.69E+10	donor2	K9#12 group for flatmount IHC, 7m8 group for DNA deep sequencing
K912-CMV-SaCas9-gRNAa/c/d	1.61E+12	7.43E+10		
7m8-CMV-SaCas9-gRNAa/c/d	4.45E+12	2.05E+11		

Supplementary Table 4 PCR primers

PCR Target name	Forward Primer 5'-3'	Reverse Primer 5'-3'
gRNA1/2-sanger	TGAGGCCACAGTCTTTCCAG	CTGACCTCTGTGATGTCCAGG
gRNA2-amplicon EZ	ACACTCTTTCCCTACACGACGCTCTTCCGATCTTTAGGCAGGGAGGTTGTAA	GACTGGAGTTCAGACGTGTGCTCTTCCGATCTTGCCCTCCATTCTGTCTCT
gRNAa/b-amplicon EZ	ACACTCTTTCCCTACACGACGCTCTTCCGATCTGCATGCTAGTGGGGTTG	GACTGGAGTTCAGACGTGTGCTCTTCCGATCTAGACAGTCAGAATCCAGCAC
gRNAa/c/d-human	TAAGTCTTAGCAAGGTGGCGG	TAAGACCCAAAGGCTGGACG
gRNAa/c/d-rhesus	TAAGTCTTAGCAAGGTGGCGG	GCTCAGTGTGATCCGACCTC
gRNAg/h-amplicon EZ	ACACTCTTTCCCTACACGACGCTCTTCCGATCTGTATTAGGGAACCCCATTCTC	GACTGGAGTTCAGACGTGTGCTCTTCCGATCTGACACTTGGTGATAAAACCATAA

*Underlined: partial illumina adaptor sequences

Supplementary Table 5 QPCR primers

qPCR Target	Forward Primer 5'-3'	Reverse Primer 5'-3'
Mouse PRPF31	GACAGCAAGATGTTTGCTGAGA	TGGCATCCACAATGACTCGG
Mouse GAPDH	GGAGAGTGTTTCCTCGTCCC	ATGAAGGGGTCGTTGATGGC
Human PRPF31[1]	CAGCAAGCAAGCCAAAGCT	CCGGATGAACTTATGGATGATG
Human GAPDH[2]	GACAGTCAGCCGCATCTTCT	TTAAAAGCAGCCCTGGTGAC

Supplementary Table 6 Antibodies

**Primary Antibodies for Immunohistochemistry**				
**Name**	**Diluton**	**Host**	**Resources**	**Catalog number**
Anti-PKCα	1:300	Mouse	Santa Cruz	sc-8393
Anti-PAX6	1:1000	Rabbit	Active Motif	61611
Anti-HA tag	1:400	Rabbit	Abcam	ab9110
1D4	50 ug/ul	Mouse	Donated by Krzysztof Palczewski Lab	
PNA	1:200	-	Thermo Fisher Scientific	L32460
Anti-RPE65	1:400	Rabbit	Abcam	ab231782
Anti-GFAP	1:500	Rabbit	Agilent Technologies	Z0334
**Primary Antibodies for Western Blotting**				
**Name**	**Dilution**	**Host**	**Resources**	**Catalog number**
Anti-HA tag	1:5000	Mouse	Thermo Fisher Scientific	26183
Anti-PRPF31	1:1000	Rabbit	Abcam	ab188577
Anti-GAPDH	1:3000	Mouse	Thermo Fisher Scientific	MA5-15738
**Secondary Antibodies**				
**Name**	**Dilution**		**Resources**	**Catalog Number**
Goat anti-Mouse IgG (H+L) Cross-Adsorbed Secondary Antibody, Alexa Fluor 488	1:1000		Thermo Fisher Scientific	A11001
Goat anti-Mouse IgG (H+L) Cross-Adsorbed Secondary Antibody, Alexa Fluor 555	1:1000		Thermo Fisher Scientific	A21422
Goat anti-Rabbit IgG (H+L) Highly Cross-Adsorbed Secondary Antibody, Alexa Fluor Plus 488	1:1000		Thermo Fisher Scientific	A32731
Goat anti-Rabbit IgG (H+L) Cross-Adsorbed Secondary Antibody, Alexa Fluor 555	1:1000		Thermo Fisher Scientific	A21428
Goat anti-Rabbit IgG (H+L) Cross-Adsorbed Secondary Antibody, Alexa Fluor 647	1:1000		Thermo Fisher Scientific	A21244
Mouse anti-rabbit IgG mAb, HRP conjugate	1:10000		Cell Signaling Technology	5127S
Rabbit anti-mouse IgG mAb, HRP conjugate	1:10000		Cell Signaling Technology	58802S

Uncropped images from Supplementary Figure 8.

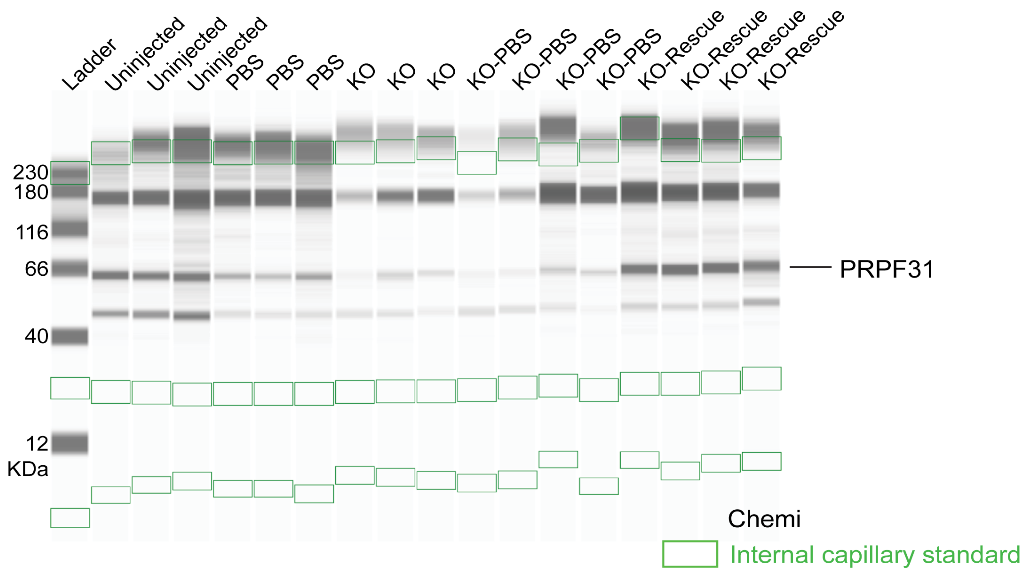

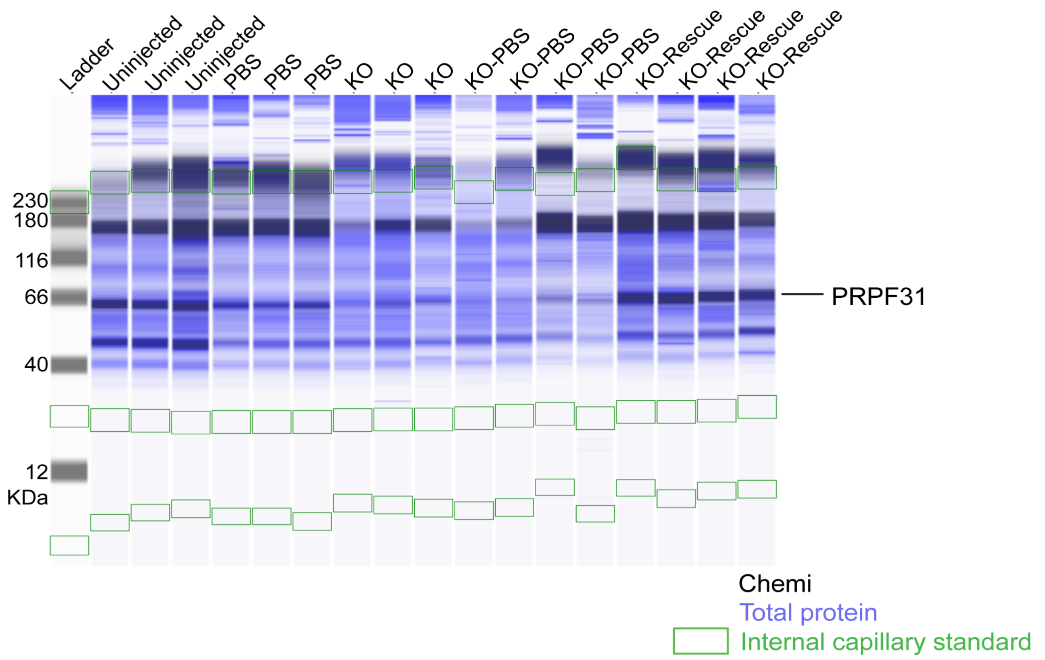

Supplementary material references

1 Rio Frio, T., Civic, N., Ransijn, A., Beckmann, J. S. & Rivolta, C. Two trans-acting eQTLs modulate the penetrance of PRPF31 mutations. *Hum Mol Genet* **17**, 3154-3165 (2008). https://doi.org:10.1093/hmg/ddn212

2 Liu, L. L. *et al.* Identification of valid reference genes for the normalization of RT-qPCR expression studies in human breast cancer cell lines treated with and without transient transfection. *PLoS One* **10**, e0117058 (2015). https://doi.org:10.1371/journal.pone.0117058
