# Supplementary material for: Gene augmentation prevents retinal degeneration in a CRISPR/Cas9-based mouse model of PRPF31 retinitis pigmentosa
Source: Nat Commun. 2022 Dec 13;13:7695. doi: 10.1038/s41467-022-35361-8 (PMC9744804; doi:10.1038/s41467-022-35361-8)
Supplement: Supplementary file 3 — Description of Additional Supplementary Files [file 41467_2022_35361_MOESM3_ESM.pdf]

# Description of Additional Supplementary Files

**Supplementary Data 1:** Statistical analyses from figures.
