# Supplementary material for: Gene augmentation prevents retinal degeneration in a CRISPR/Cas9-based mouse model of PRPF31 retinitis pigmentosa
Source: Nat Commun. 2022 Dec 13;13:7695. doi: 10.1038/s41467-022-35361-8 (PMC9744804; doi:10.1038/s41467-022-35361-8)
Supplement: Supplementary file 5 — Reporting Summary [file 41467_2022_35361_MOESM5_ESM.pdf]

nature portfolio

Corresponding author(s): Leah C. Byrne

Last updated by author(s): Nov 3, 2022

Reporting Summary

Nature Portfolio wishes to improve the reproducibility of the work that we publish. This form provides structure for consistency and transparency in reporting. For further information on Nature Portfolio policies, see our Editorial Policies and the Editorial Policy Checklist.

Statistics

For all statistical analyses, confirm that the following items are present in the figure legend, table legend, main text, or Methods section.

n/a	Confirmed	
☐	☒	The exact sample size (*n*) for each experimental group/condition, given as a discrete number and unit of measurement
☐	☒	A statement on whether measurements were taken from distinct samples or whether the same sample was measured repeatedly
☐	☒	The statistical test(s) used AND whether they are one- or two-sided<br>*Only common tests should be described solely by name; describe more complex techniques in the Methods section.*
☐	☒	A description of all covariates tested
☐	☒	A description of any assumptions or corrections, such as tests of normality and adjustment for multiple comparisons
☐	☒	A full description of the statistical parameters including central tendency (e.g. means) or other basic estimates (e.g. regression coefficient) AND variation (e.g. standard deviation) or associated estimates of uncertainty (e.g. confidence intervals)
☐	☒	For null hypothesis testing, the test statistic (e.g. *F*, *t*, *r*) with confidence intervals, effect sizes, degrees of freedom and *P* value noted<br>*Give P values as exact values whenever suitable.*
☒	☐	For Bayesian analysis, information on the choice of priors and Markov chain Monte Carlo settings
☒	☐	For hierarchical and complex designs, identification of the appropriate level for tests and full reporting of outcomes
☒	☐	Estimates of effect sizes (e.g. Cohen's *d*, Pearson's *r*), indicating how they were calculated

Our web collection on statistics for biologists contains articles on many of the points above.

Software and code

Policy information about availability of computer code

Data collection	No custom code was used in the collection of data. ERGs were recorded using Diagnosys software V6.64. OCT imaging was collected using Bioptogen 3.0.8 software. The thickness of retinal layers was measured from B-scan images using Image J v1.51, and the retinal thickness heatmaps were plotted from volume scans with Bioptigen InVivoVue 3.0.8 software.
Data analysis	All data analysis tools have been previously published and are publicly available. For SpCas9, gRNA sequences were designed using the Synthego Knockout Guide Designer (https://design.synthego.com/#/). gRNAs for SaCas9 were designed using CHOPCHOP (https://chopchop.cbu.uib.no/) and CRISPOR (http://crispor.tefor.net/). For in vivo editing efficiency analysis, sequencing was performed by Genewiz (https://www.genewiz.com/). For plots, Cutadapt2.10 was used to trim adapter sequences. Editing rates were analyzed and visualized using CRISPRESSO (https://crispresso.pinellolab.partners.org/) For differential expression analysis, a NextSeq 500 Illumina sequencer with high output 150 cycles was used to obtain 2 x 75 paired-end reads. We performed quality control of raw Fastq files with FastQC software Version 0.11.9, and Cutadapt2.10 was used to trim adapter sequences and eliminate all low-quality reads below the Phred score cut-off of 20. Salmon 1.1.0 was used to index and quantify transcripts using a mm10 Salmon index, which was produced with salmon index using a partial selective alignment method. Differential gene expression was analyzed using DESeq2 (2.11.40.5). For Jess SimpleWesterns, analysis was performed using Compass software v6.1.0 (BioTechne).

For manuscripts utilizing custom algorithms or software that are central to the research but not yet described in published literature, software must be made available to editors and reviewers. We strongly encourage code deposition in a community repository (e.g. GitHub). See the Nature Portfolio guidelines for submitting code & software for further information.

Data

Policy information about availability of data

All manuscripts must include a data availability statement. This statement should provide the following information, where applicable:

- Accession codes, unique identifiers, or web links for publicly available datasets
- A description of any restrictions on data availability
- For clinical datasets or third party data, please ensure that the statement adheres to our policy

Deep sequencing and genome editing quantification data generated in this study have been deposited in Dryad (https://doi.org/10.5061/dryad.bcc2fqzf1). FastQ files are publicly available. Source data are provided with this paper.

Field-specific reporting

Please select the one below that is the best fit for your research. If you are not sure, read the appropriate sections before making your selection.

- [x] Life sciences
- [ ] Behavioural & social sciences
- [ ] Ecological, evolutionary & environmental sciences

For a reference copy of the document with all sections, see nature.com/documents/nr-reporting-summary-flat.pdf

Life sciences study design

All studies must disclose on these points even when the disclosure is negative.

Sample size	Whenever possible, sample sizes were determined according to the experiment and protocol, taking into account power, effect size and significance. Consideration of samples sizes was determined with advice from the University of Pittsburgh Biostatistics core. Experiments were originally designed to provide sufficient power (80%) to detect a difference with an effect size ranging between 1.08 and 3. In some cases, such as primate and human retinal tissue experiments, and for some mouse litters, the availability of samples or offspring was a determining factor in sample size.
Data exclusions	No data was excluded from analysis.
Replication	Reproducibility of experimental findings was verified through the use of multiple, validating experiments utilizing alternative approaches. For example, PRPF31 knockout was performed using SaCAs9 and SpCas9 constructs. Retinal degeneration was observed in animals treated with SaCas9 and SpCas9 constructs across species. These experiments cross-validate the findings regarding the effects of CRISPR editing of Prpf31. Each unique experiment was performed once. Details including the number of biological replicates are available for each experiment in the manuscript.
Randomization	Animals were assigned into experimental groups randomly, with approximately equal numbers of male and female mice represented in each group, as possible, depending on the composition of litters. For non-human primate and human tissue, samples were not assigned to groups. Single biological replicates were used in the order they became available. The control of covariates are not relevant to human and primate samples.
Blinding	In some experiments (deep sequencing genome editing analysis, differential expression analysis) one investigator was responsible for animal injections, and a blinded investigator performed data collection and analysis. For other experimental procedures (ERGs, OCTs, fundus imaging), due to a shortage of personnel and limited laboratory access during the Covid-19 pandemic, in some experiments, one researcher performed injections as well as data collection. In these cases, The PIs discussed data collection and analysis prior to and after collection with the researcher and methods and results were thoroughly reviewed by the principal investigator.

Reporting for specific materials, systems and methods

We require information from authors about some types of materials, experimental systems and methods used in many studies. Here, indicate whether each material, system or method listed is relevant to your study. If you are not sure if a list item applies to your research, read the appropriate section before selecting a response.

Materials & experimental systems

n/a	Involved in the study	
☐	☒	Antibodies
☐	☒	Eukaryotic cell lines
☒	☐	Palaeontology and archaeology
☐	☒	Animals and other organisms
☐	☒	Human research participants
☒	☐	Clinical data
☒	☐	Dual use research of concern

Methods

n/a	Involved in the study	
☒	☐	ChIP-seq
☒	☐	Flow cytometry
☒	☐	MRI-based neuroimaging

Antibodies

Antibodies used

Antibodies are provided in Supp Table 6:

Primary Antibodies for Immunohistochemistry:
Name Diluton Host Resources Catalog number
Anti-PKCα 1:300 Mouse Santa Cruz sc-8393
Anti-PAX6 1:1000 Rabbit Active Motif 61611
Anti-HA tag 1:400 Rabbit Abcam ab9110
1D4 50 ug/ul Mouse Donated by Krzysztof Palczewski Lab
PNA 1:200 - Thermo Fisher Scientific L32460
Anti-RPE65 1:400 Rabbit Abcam 231782
Anti-GFAP 1:500 Rabbit Agilent Technologies Z0334

Primary Antibodies for Western Blotting
Name Dilution Host Resources Catalog number
Anti-HA tag 1:5000 Mouse Thermo Fisher Scientific 26183
Anti-PRPF31 1:1000 Rabbit Abcam 188577
Anti-GAPDH 1:3000 Mouse Thermo Fisher Scientific MA5-15738

Secondary Antibodies
Name Dilution Resources Catalog Number
Goat anti-Mouse IgG (H+L) Cross-Adsorbed Secondary Antibody, Alexa Fluor 488 1:1000 Thermo Fisher Scientific A11001
Goat anti-Mouse IgG (H+L) Cross-Adsorbed Secondary Antibody, Alexa Fluor 555 1:1000 Thermo Fisher Scientific A21422
Goat anti-Rabbit IgG (H+L) Highly Cross-Adsorbed Secondary Antibody, Alexa Fluor Plus 488 1:1000 Thermo Fisher Scientific A32731
Goat anti-Rabbit IgG (H+L) Cross-Adsorbed Secondary Antibody, Alexa Fluor 555 1:1000 Thermo Fisher Scientific A21428
Goat anti-Rabbit IgG (H+L) Cross-Adsorbed Secondary Antibody, Alexa Fluor 647 1:1000 Thermo Fisher Scientific A21244
Mouse anti-rabbit IgG mAb, HRP conjugate 1:10000 Cell Signaling Technology 5127S
Rabbit anti-mouse IgG mAb, HRP conjugate 1:10000 Cell Signaling Technology 58802S

Validation

Validation information for primary antibodies can be found at the websites or articles listed below.

IHC:
Anti-PKCa: https://datasheets.scbt.com/sc-8393.pdf
Anti-PAX6: https://www.thermofisher.com/antibody/product/PAX6-Antibody-Polyclonal/61611
Anti-HA tag: https://www.abcam.com/ha-tag-antibody-chip-grade-ab9110.html
1D4: https://pubmed.ncbi.nlm.nih.gov/6529569/
PNA: https://www.thermofisher.com/order/catalog/product/L21409
Anti-RPE65: https://www.abcam.com/rpe65-antibody-epr22579-44-ab231782.html
Anti-GFAP: https://www.agilent.com/en/product/immunohistochemistry/antibodies-controls/primary-antibodies/glial-fibrillary-acidic-protein-%28concentrate%29-76683

Western blotting:
Anti-HA tag: https://www.thermofisher.com/antibody/product/HA-Tag-Antibody-clone-2-2-2-14-Monoclonal/26183
Anti-PRPF31: https://www.abcam.com/prpf31-antibody-epr14587-ab188577.html
Anti-GAPDH: https://www.thermofisher.com/antibody/product/GAPDH-Loading-Control-Antibody-clone-GA1R-Monoclonal/MA5-15738-HRP

Eukaryotic cell lines

Policy information about cell lines

Cell line source(s)

HEK293 AAV293 cells (Cell Biolabs) were used for AAV packaging and for in vitro experiments.

Authentication

AAV923 cells are passaged fewer than 5 times after purchase and are regularly authenticated by morphology.

Mycoplasma contamination

These cells tested negative for mycoplasma contamination.

Commonly misidentified lines
(See ICLAC register)

There are no commonly misidentified lines used in the present study.

Animals and other organisms

Policy information about studies involving animals; ARRIVE guidelines recommended for reporting animal research

Laboratory animals	Mice. C57BL/6J mice and Cas9-expressing mice (H11Cas9 CRISPR/Cas9 knock-in mice, B6J.129(Cg)-Igs2<tm1.1(CAG-cas9*)Mmw>/J, Stock# 028239) were purchased from Jackson Laboratories. Mice used for systemic injections were neonates. Mice used for intravitreal and subretinal injections were 4-5 weeks old.<br><br>Rhesus Macaque. A 5-year-old female was euthanized for other purposes, and the eyes were enucleated at that point.
Wild animals	The study did not involve wild animals.
Field-collected samples	The study did not involve sample collected from the field.
Ethics oversight	All procedures were performed in accordance with the Association for Research in Vision and Ophthalmology statement for the Use of Animals in Ophthalmic and Vision Research. All animal experiments were approved by the University of Pittsburgh Institutional Animal Care and Use Committee (IACUC #21058910). Human tissue. All experiments were performed with approval and oversight from the University of Pittsburgh Committee for Oversight of Research and Clinical Training Involving Decedents (CORID, approval #927). Eyes from donors was obtained from the Center for Organ Recovery & Education (CORE). Organ recovery was performed by CORE and consent was obtained by CORE. No compensation was provided to donors for this study.

Note that full information on the approval of the study protocol must also be provided in the manuscript.

Human research participants

Policy information about studies involving human research participants

Population characteristics	Eyes from donors was obtained through the Center for Organ Recovery & Education. Donor #1, 45-year-old male; donor #2, 32-year-old male, no history of retinal disease.
Recruitment	Organ donors were not recruited. Tissue was obtained through the Center for Organ Recovery and Education (CORE).
Ethics oversight	All experiments were performed with approval and oversight from the University of Pittsburgh Committee for Oversight of Research and Clinical Training Involving Decedents (CORID, approval #927). Eyes from donors was obtained from the Center for Organ Recovery & Education (CORE). Organ recovery was performed by CORE and consent was obtained by CORE. No compensation was provided to donors for this study.

Note that full information on the approval of the study protocol must also be provided in the manuscript.
